# Cardiomyocytes Derived from Induced Pluripotent Stem Cells as a Disease Model for Propionic Acidemia

**DOI:** 10.3390/ijms22031161

**Published:** 2021-01-25

**Authors:** Esmeralda Alonso-Barroso, Belén Pérez, Lourdes Ruiz Desviat, Eva Richard

**Affiliations:** 1 Centro de Biología Molecular Severo Ochoa UAM-CSIC, Universidad Autónoma de Madrid, 28049 Madrid, Spain; esmeralda.alonso@cbm.csic.es (E.A.-B.); bperez@cbm.csic.es (B.P.); lruiz@cbm.csic.es (L.R.D.); 2 Centro de Diagnóstico de Enfermedades Moleculares (CEDEM), 28049 Madrid, Spain; 3 Centro de Investigación Biomédica en Red de Enfermedades Raras (CIBERER), ISCIII, 28029 Madrid, Spain; 4 Instituto de Investigación Sanitaria Hospital La Paz (IdiPaz), ISCIII, 28029 Madrid, Spain

**Keywords:** propionic acidemia, cardiac dysfunction, iPSC, iPSC-derived cardiomyocytes, disease model

## Abstract

Propionic acidemia (PA), one of the most frequent life-threatening organic acidemias, is caused by mutations in either the *PCCA* or *PCCB* genes encoding both subunits of the mitochondrial propionyl-CoA carboxylase (PCC) enzyme. Cardiac alterations (hypertrophy, dilated cardiomyopathy, long QT) are one of the major causes of mortality in patients surviving the neonatal period. To overcome limitations of current cellular models of PA, we generated induced pluripotent stem cells (iPSCs) from a PA patient with defects in the *PCCA* gene, and successfully differentiated them into cardiomyocytes. PCCA iPSC-derived cardiomyocytes exhibited reduced oxygen consumption, an accumulation of residual bodies and lipid droplets, and increased ribosomal biogenesis. Furthermore, we found increased protein levels of HERP, GRP78, GRP75, SIG-1R and MFN2, suggesting endoplasmic reticulum stress and calcium perturbations in these cells. We also analyzed a series of heart-enriched miRNAs previously found deregulated in the heart tissue of a PA murine model and confirmed their altered expression. Our novel results show that PA iPSC-cardiomyocytes represent a promising model for investigating the pathological mechanisms underlying PA cardiomyopathies, also serving as an ex vivo platform for therapeutic evaluation.

## 1. Introduction

To date, there is no cure for organic acidemias/acidurias (OAs), which are a group of rare inherited metabolic diseases characterized by the excessive accumulation of organic acids in body fluids [1]. OAs are severe diseases that can affect multiple organ systems; however, many of them cause cardiac dysfunction. The most frequent life-threatening OA that is also strongly associated with cardiac complications is propionic acidemia (PA, MIM#606054), which is caused by the deficiency of propionyl-CoA carboxylase (PCC). Cardiac complications account for significant morbidity and mortality in PA patients, most commonly in the form of cardiomyopathy and prolonged QT intervals [2,3]. Whereas cardiomyopathy seems to appear mostly during childhood (mean onset age 7 years) [4], long QT syndrome observed in PA has been shown to be progressive during aging. The available evidence suggests that dilated cardiomyopathy and prolonged QT are a secondary consequence of PCC dysfunction. No specific biochemical marker related to cardiomyopathy development has to date been identified. The ultimate mechanism for cardiac alterations in PA remains unclear and is likely multifactorial.

PA is caused by mutations in either the *PCCA* or *PCCB* genes, encoding both subunits of biotin-dependent PCC, a mitochondrial enzyme that catalyzes the carboxylation of propionyl-CoA to d-methylmalonyl-CoA, which eventually enters the Krebs cycle as succinyl-CoA. Propionyl-CoA is common to the pathway for degradation of some amino acids (isoleucine, valine, threonine and methionine), odd-chain fatty acids and cholesterol. Gut bacteria also represent an important source of propionate. Biochemically, this disorder is characterized by the accumulation of propionyl-CoA, considered as the major toxic agent, and metabolites of alternative propionate oxidation.

The study of the mechanisms involved in disease physiopathology has been mainly performed using the hypomorphic PA mouse model that mimics the biochemical and clinical phenotype [5]. Using this model, bioenergetic failure, oxidative damage and deregulation of miRNAs induced by accumulating propionyl-CoA have been described as potential mechanisms contributing to PA physiopathology [6,7,8]. The limitations of animal models for the study of cardiac energy metabolism [9] and of the commonly available cellular human models such as fibroblasts, underline the importance of generating new relevant cell models to provide deeper insight into the underlying mechanisms of disease. The use of in vitro models with human cellular context is highly recommended and, in this sense, induced pluripotent stem cells (iPSCs) have certain advantages since they provide the genetic background of the patient and represent an unlimited source of biological material for the study of pathophysiology and treatment effectiveness [10]. We have previously generated an iPSC line from a PA patient with defects in the *PCCA* gene that showed full pluripotency, differentiation capacity and genetic stability [11].

In the present study, we aimed to establish a platform that served as a disease model to study the cellular and molecular alterations operating in cardiac tissue affected by PA disease. We described the characterization of cardiomyocytes derived from the *PCCA* iPSC line (*PCCA* iPSC-CMs) and the analysis of specific pathways potentially involved in cardiac PA physiopathology.

## 2. Results and Discussion

PA disorder primarily affects the heart, among other organ systems. The hypomorphic PA mouse model has provided a good model for the study of PA physiopathology, allowing the characterization of some aspects related to the cardiac phenotype [6,7,8,12]. However, murine models present some limitations since a mouse heart differs considerably from a human one, both at the structural level [13] and in physiological parameters, such as heart rate (400–600 beats/minute in mice), calcium handling and ionic currents [14]. In addition, the difficulty in obtaining a single model for all the genotypes observed in OAs, with the aim of developing a personalized medicine approach, has prompted the generation of new patient-specific models of disease. In this context, iPSCs offer unprecedented opportunities since these cells can be directed to become any cell type in the body [15]. iPSC-derived cardiomyocytes (iPSC-CMs) represent a good cellular model in which disease mechanisms can be experimentally deciphered in a human context [16]. A significant number of PA patients develop cardiac complications, and available evidence suggests that this cardiac dysfunction is mainly driven by the accumulation of toxic metabolites [2,3,17,18]. However, the mechanistic basis underlying this dysfunction has yet to be fully elucidated, although several pathogenic mechanisms have been proposed, including mitochondrial dysfunction resulting in bioenergetic deficiency and oxidative damage, miRNA deregulation modifying signaling pathways and alteration in post-translational modifications of histones [19]. In this work, we investigated several parameters related to the above-described processes in cardiomyocytes differentiated from patient-derived iPSCs to validate them as PA disease models for the investigation of PA associated cardiac dysfunction.

**Figure 1:**
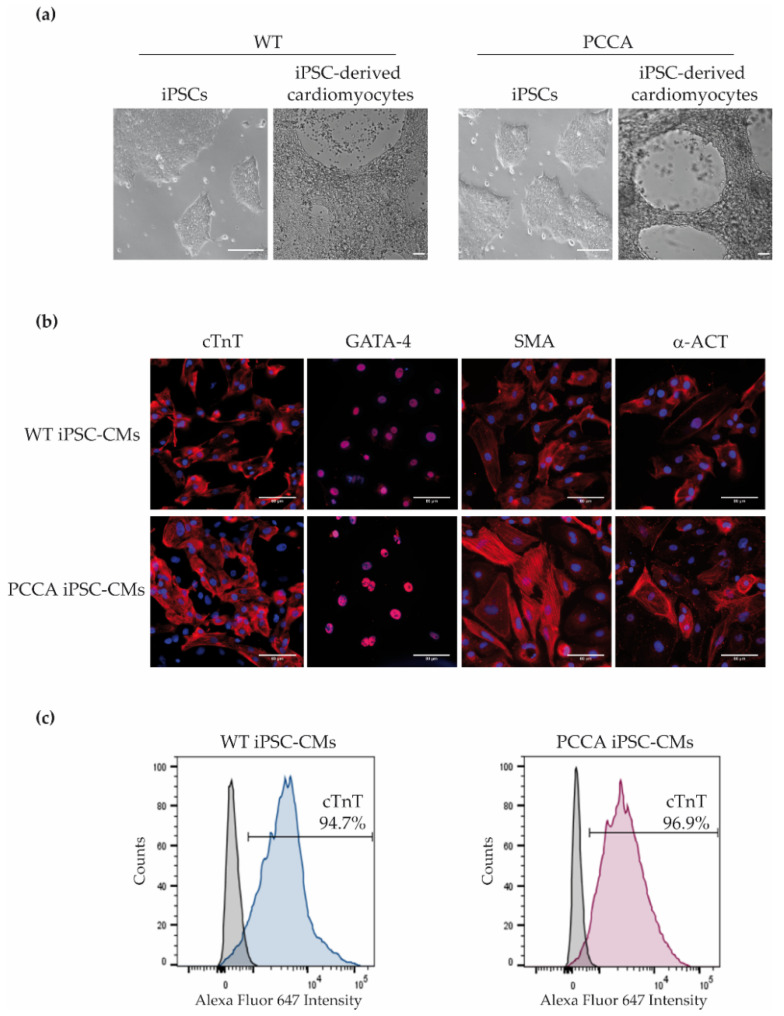
Phase-contrast pictures of iPSCs and differentiated cardiomyocytes and expression of cardiac markers in wild-type (WT) and *PCCA* iPSC-derived cardiomyocytes (iPSC-CMs). (**a**) Phase-contrast images of iPSC and iPSC-derived cardiomyocytes of WT and *PCCA*; scale bar: 100 µm. (**b**) Immunofluorescence analysis for cardiac troponin T (cTnT), GATA-4, α-smooth muscle actin (SMA) and α-actinin (α-ACT) in iPSC-derived cardiomyocytes; scale bar: 80 µm. (**c**) Flow cytometry analysis for cTnT cardiac marker. A representative experiment for cTnT expression is shown.

We previously generated a PA-derived iPSC line carrying mutations in the *PCCA* gene: c.1899+4_1899+7delAGTA; p.(Cys616_Val633del) and c.1430−?_1643+?del; p.(Gly477Glufs*9) (Figure 1a) [11]. This patient with a mild form of the disease presented developmental delay, hypotonia, recurrent infections and chronic pancreatitis, and to date, presented no cardiac alterations. We compared this line with a wild-type (WT) iPSC line derived from a healthy unrelated individual without cardiovascular disease and with normal PCC expression. We recognize that the current gold standard is to compare patient samples to gene-corrected isogenic controls, usually made by CRISPR-Cas9 gene-editing technology; however, in our study, we use an age-matched control because the *PCCA* patient is compound heterozygous with a large deletion in one allele.

The *PCCA* and WT iPSC lines both generated cardiomyocytes with spontaneous beating activity and high cardiac differentiation efficiency, and with no significant difference between the lines in the differentiation process. Phase-contrast images of differentiated cardiomyocytes are shown in Figure 1a. WT and *PCCA* iPSC-CMs expressed cardiac-specific markers, cardiac troponin T (cTnT), α-smooth muscle actin (SMA), GATA4 and α-actinin (α-ACT) (Figure 1b). Positive cells for cTnT expression were confirmed by flow cytometry, and approximately 90–95% of the control and patient cells were cTnT+ (Figure 1c). We confirmed the presence of increased propionylcarnitine levels (3.43 µM in *PCCA* iPSC-CMs versus 0.05 µM in WT iPSC-CMs), a biochemical hallmark of the disease, as well as the absence of *PCCA* protein in *PCCA* iPSC-CMs.

**Figure 2:**
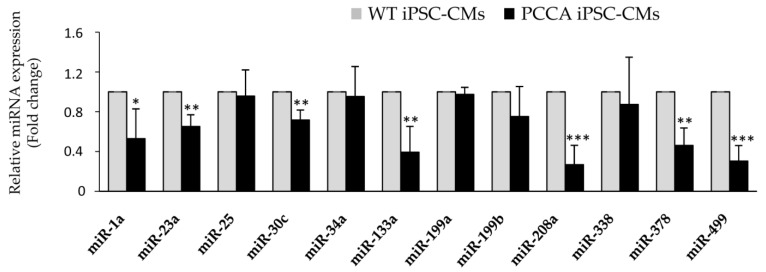
Analysis of miRNA expression in WT and *PCCA* iPSC-CMs. Relative expression levels of miR-1a, miR-23a, miR-25, miR-30c, miR-34a, miR-133a, miR-199a, miR-199b, miR-208a, miR-338, miR-378 and miR-499 are evaluated by qRT-PCR in iPSC-derived cardiomyocytes. Data represents mean ± standard deviation of three independent cardiomyocyte differentiation triplicates at least. Statistical significance is determined by the Student’s *t*-test. * *p* < 0.05; ** *p* < 0.01; *** *p* < 0.001.

In our study, we aimed to analyze several altered pathways previously identified in heart tissue of the hypomorphic PA mouse model and potentially related to its cardiac phenotype [6,8,12]. First, we evaluated the expression levels of a series of 12 cardiac-enriched miRNAs known to play an important role in cardiac development, dysfunction and failure [8,12]. We confirmed a significant downregulation in seven of them (miR-1a, miR-23a, miR-30c, miR-133a, miR-208a, miR-378 and miR-499) in *PCCA* iPSC-CMs compared to WT cardiomyocytes (Figure 2). Models of heart failure are characterized by a decrease in the expression of miR-1a, miR-133a and miR-30c, among others, which play vital roles in the maintenance of heart histology and function [20,21]. Our results suggest that, in PA cardiomyocytes, the downregulation of these miRNAs may be responsible, at least in part, for cardiac dysfunction as they exert a cardioprotective role [22,23]. Most of our results in PA iPSC-derived cardiomyocytes coincided with those obtained in plasma samples from PA patients, where the cardiomiRs were found to be downregulated [8,12]. Specifically, miR-133a, among others, was highly reduced in plasma of the *PCCA* patient studied in this work (unpublished data). Results from cardiomyocytes and plasma patients differed from those obtained in the heart of the murine model, in which these miRNAs appeared to have increased [8]. These differences may have likely been due to the different miRNA gene regulation mechanisms in mice and humans [24] or to developmental stage-specific differences, as in PA mice, these miRNAs showed maximal expression in the heart at 5 months of age, while at earlier or later ages, variable or no relative differences were observed.

**Figure 3:**
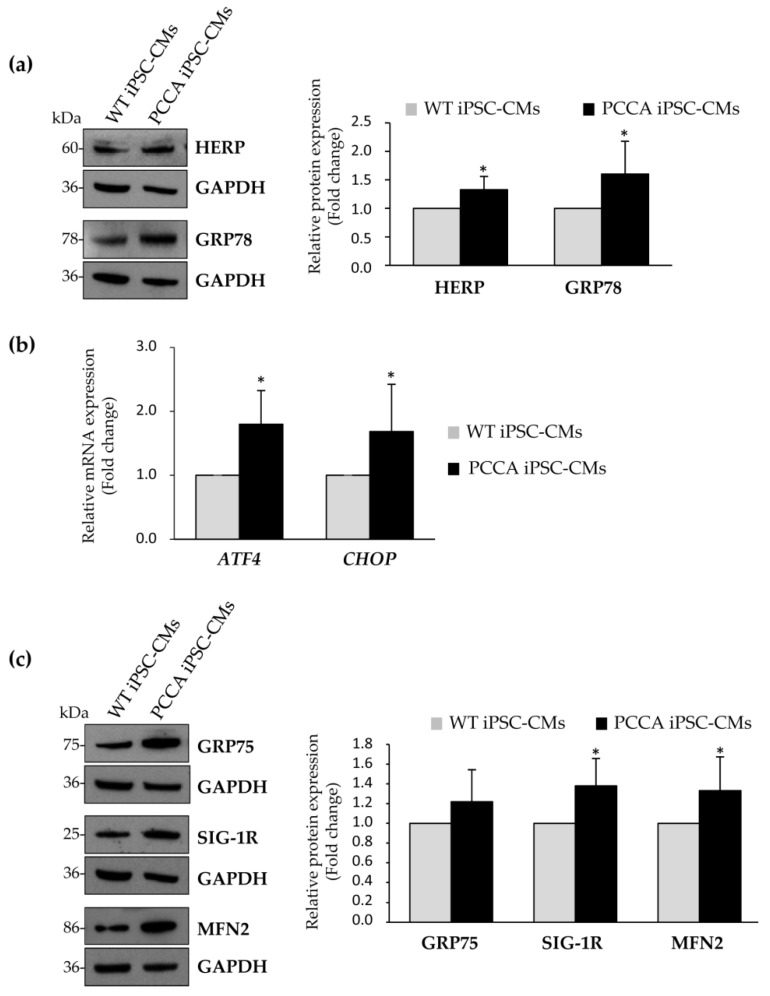
Evaluation of protein and mRNA levels involved in the unfolded protein response (UPR) and mitochondria-associated membranes (MAMs) in WT and *PCCA* iPSC-CMs. (**a**,**b**) Expression analysis of several proteins involved in the UPR by western blot (**a**) or by qRT-PCR (**b**). (**c**) Analysis of protein levels of MAMs proteins by western blot. In panels (**a**,**c**), representative blots and the corresponding quantification of proteins by laser densitometry are shown as the mean ± standard deviation of at least three experiments. In each blot, GADPH is used as a loading control. In (**b**), data represents the mean ± standard deviation of at least three independent cardiomyocyte differentiation triplicates. Statistical significance is determined by Student’s *t*-test. * *p* < 0.05.

It is interesting to note that miR-378 acts as a negative regulator of the endoplasmic reticulum (ER) stress response, among other functions [25]. Thus, its downregulation could be responsible, in part, for an increase of ER stress in iPSC-derived cardiomyocytes. To investigate this hypothesis, we next analyzed the levels of some proteins and genes involved in ER unfolded protein response (UPR) and the levels of several proteins that reside at mitochondria-associated membranes (MAMs). ER and mitochondria interact at MAMs to exchange lipids and calcium and regulate cellular homeostasis [26]. The expression study of several proteins involved in UPR showed an increase in homocysteine-inducible ER stress protein (HERP), 78 kDa glucose-regulated protein (GRP78) levels (Figure 3a) and *ATF4* and *CHOP* mRNA levels in *PCCA* cells compared to the control ones (Figure 3b). GRP78 is a potential target of miR-378 and the increased expression of GRP78 protein in our model could be explained, in part, by the downregulation of this miRNA [27]. In addition, our results showed increased levels of the MAMs 75 kDa glucose-regulated protein (GRP75), sigma-1 receptor (SIG-1R) and mitofusin-2 (MFN2) proteins in *PCCA* iPSC-CMs in comparison with WT iPSC-CMs (Figure 3c). The structural and functional interactions between the ER and mitochondria are essential for normal cardiac function and alterations in the amount, structure or function of MAMs have been related to cardiovascular diseases [28]. Elevated expression of the ER stress markers GRP78, eIF2α and XBP1, and increased activation of the UPR, has been observed in patients with inherited dilated cardiomyopathy [29]. Recently, we described impaired calcium handling related to SERCA2a protein dysfunction in the hypomorphic PA mouse model, which could lead to cardiac dysfunction and ventricular arrhythmias [7]. The observed alterations in the amount of UPR and MAMs proteins in PA patient iPSC-CMs suggest the presence of ER stress and alterations in calcium homeostasis, and these results will be followed up with further studies focused on in vivo calcium imaging in iPSC-CMs.

**Figure 4:**
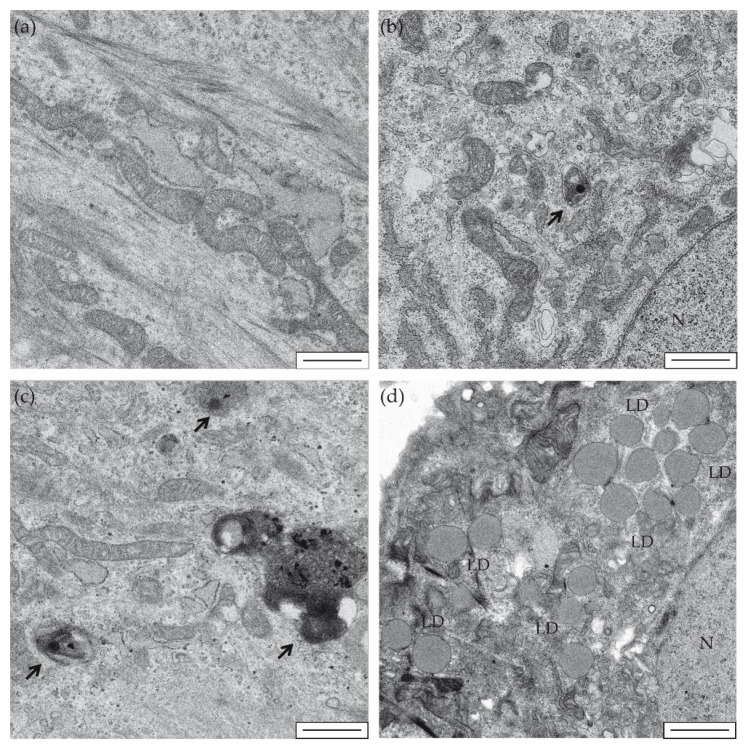
Electron microscopy of iPSC-derived cardiomyocytes. Representative images are shown of WT-iPSC-CMs (**a**,**b**) and *PCCA* iPSC-CMs (**c**,**d**) at 5000× magnification. Black arrows show degradation vesicles (**b**,**c**). LD: lipid droplets (**d**). N: cell nucleus (**b**,**d**). Scale bar: 1 µm.

Taking into account that MAMs were also linked to autophagy, which was found inhibited in hearts of the PA mouse model [8], here, we aimed to analyze this cellular process by electron microscopy in iPSC-derived cardiomyocytes (Figure 4). Our study showed an increase in the number and size of vesicles with degradation material in *PCCA* iPSC-CMs compared to WT iPSC-CMs (Figure 4a–c). These residual bodies may be products of lysosomal digestion that accumulate indigestible materials, which could suggest an alteration in the process of autophagy in these cells. In addition, *PCCA* iPSC-CMs displayed a large increase in the number and size of lipid droplets (Figure 4a,b,d). The inclusion of many lipid droplets has also been observed in muscle biopsies of PA patients [30] and in the liver of the *Pcca* knockout mouse model of PA [31], possibly indicating impaired β-oxidation.

**Figure 5:**
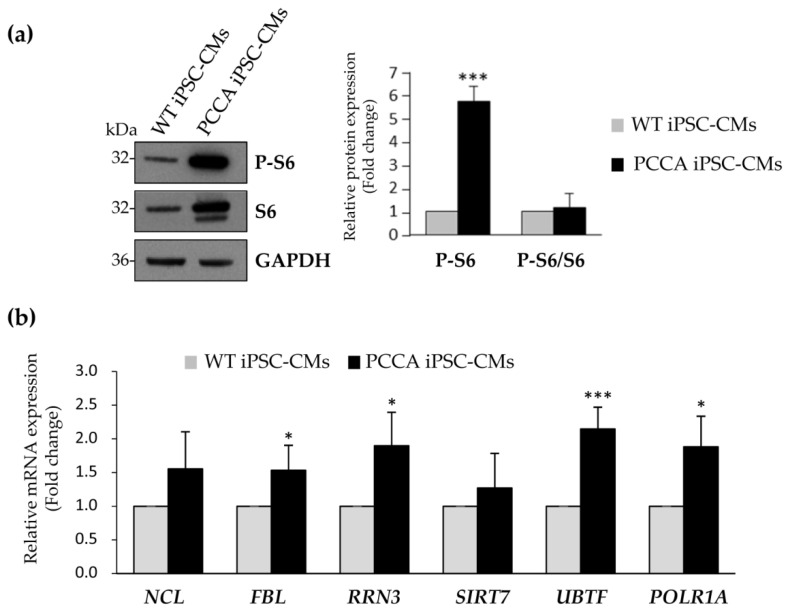
Analysis of expression levels of proteins involved in ribosomal biogenesis. (**a**) Representative blot of the analysis of S6 ribosomal protein and its phosphorylated form. GADPH is used as loading control. The corresponding quantification by laser densitometry is shown as the mean ± standard deviation of at least three experiments. (**b**) Relative mRNA expression of *NCL*, *FBL*, *RRN3*, *SIRT7*, *UBTF* and *POLR1A* genes by qRT-PCR. Data represents the mean ± standard deviation of three independent biological triplicates. Statistical significance is determined by Student’s *t*-test. * *p* < 0.05; *** *p* < 0.001.

mRNA translation in the heart occurs at relatively low levels unless signaling increases substantially and/or is sustained, which can be due to physiological or pathological conditions [32]. We next investigated the expression of several proteins involved in ribosomal biogenesis, and our results showed a significant increase of the phosphorylated form of ribosomal protein S6 levels in PA CMs compared to the controls (Figure 5a). S6 protein is a component of the 40S ribosomal subunit, and increased phosphorylation of S6 protein has been suggested as a mechanism to regulate the efficiency of mRNA translation [33]. In addition, we evaluated the expression at the mRNA level of different genes involved in ribosomal biogenesis, such as nucleolin (*NCL*), rRNA methyltransferase fibrillarin (*FBL*), Pol I-activating NAD-dependent histone deacetylase Sirtuin 7 (*SIRT7*), Pol I-specific transcription initiation factor (*RRN3*), ribosomal RNA upstream binding transcription factor (*UBTF*) and large subunit of Pol I (*POLR1A*). Figure 5b shows increased mRNA levels in all genes analyzed, being statistically significant in *FBL*, *RRN3*, *UBTF* and *POLR1A*, suggesting a transcriptional mechanism that could increase ribosomal biogenesis in *PCCA* iPSC-CMs. The perturbation of any major step in the ribosomal biogenesis process triggers ribosomal stress and leads to cell death [34], while its activation has been associated with different pathophysiological conditions, such as skeletal muscle hypertrophy [35] and heart remodeling [36]. It has been proposed that targeting protein translation pathways, especially when they are aberrantly activated in conditions of mechanical disturbance, may represent a novel therapeutic strategy to confer cardioprotection [32]. iPSC-derived PA cardiomyocytes will serve as a cellular platform to investigate whether this approach is applicable in PA disease.

**Figure 6:**
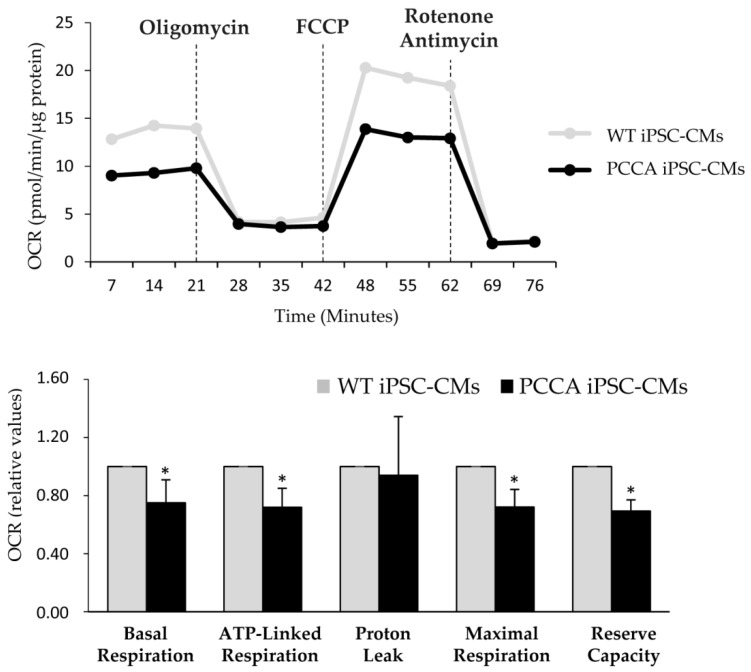
Bioenergetic profile of WT and *PCCA* iPSC-CMs. Representative profile of basal oxygen consumption rate (OCR) in WT and *PCCA* iPSC-CMs, and after the addition of oligomycin, FCCP, rotenone and antimycin A. Relative values of OCR are shown as the mean ± standard deviation of three to five wells from three independent cardiomyocyte differentiations. Statistical significance is determined by Student’s *t*-test. * *p* < 0.05.

Efficient mitochondrial function is required in tissues with high energy demand, such as cardiac tissue, and mitochondrial dysfunction has been associated with cardiovascular disease. We evaluated the bioenergetic profile in PA cardiomyocytes by Seahorse analysis upon the sequential addition of different drugs interfering with mitochondrial respiration. The results revealed a significant reduction in ATP-linked oxygen consumption ratio, and maximal oxygen consumption rate (OCR) and reserve capacity in iPSC-derived cardiomyocytes from the *PCCA* patient compared to the control, indicating a decreased oxidative phosphorylation (Figure 6). This result may be related to the metabolic change that occurs during the development of cardiac alterations and heart failure consisting in the reduction of energy production by mitochondria and an increase in anaerobic glycolysis [9]. It is worth noting that these observations are in agreement with the disturbances in mitochondrial function (i.e., inhibition of specific complexes of the electron transport chain) and redox homeostasis (increase in mitochondrial ROS, antioxidant defenses, etc.) that have been previously observed in PA mice tissues [6] and also in PA patient samples [30,37,38,39].

Our results provide evidence that several pathomechanisms may have a relevant role in cardiac dysfunction, a common complication in PA disease. It is unlikely that a single mechanism is responsible for driving heart disease in PA patients, which is likely multifactorial. The *PCCA* patient from whom the iPSC originated is not currently presenting cardiomyopathy at 13 years of age but may well develop cardiac alterations in the future since this has been described to be progressive with age. The study of more PA patients with different genotypes and cardiac phenotypes will provide a deeper understanding of these processes since other regulatory or epigenetic factors may be involved. To date, there is no evidence of the contribution of cardio risk or cardio-protective SNVs contributing to the cardiac phenotype in PA patients, but with the implementation of whole exome sequencing/whole genome sequencing for diagnostic purposes, this may be elucidated in the future.

The present study represents the first report that provides a characterization of cardiomyocytes derived from iPSCs generated by PA patient fibroblast reprogramming. This new cellular PA model offers a powerful tool to unravel disease mechanisms and, potentially, to enable drug screening/drug testing. Despite improved therapy over the past few decades, the outcome of PA patients is still unsatisfactory, highlighting the requirement to evaluate new therapies aimed at preventing or alleviating the clinical symptoms. The potential beneficial effects of antioxidant compounds have been described in PA patient-derived fibroblasts [40] and in the hypomorphic PA mouse model [41]. Our next step will be to investigate the effects of mitochondrial-targeted antioxidants such as Mito-Q, mitochondrial biogenesis activators (PPAR agonists such as pioglitazone or bezafibrate), for improving mitochondrial function. Additional research is also required to determine whether the mechanisms identified in this work are indeed responsible for the cardiac phenotype and will help in formulating better personalized therapeutic strategies in the future.

## 3. Materials and Methods

### 3.1. Cell Lines

The iPSC lines used in this work were: (i) a *PCCA* deficient iPSC line (PCCA23-FiPS4F6 or UAMi001-A) generated by reprogramming of patient-derived fibroblasts with defects in the *PCCA* gene (c.1899+4_1899+7delAGTA; p.(Cys616_Val633del) and c.1430-?_1643+?del; p.(Gly477Glufs*9)) using Sendai virus [11]; and (ii) a healthy control iPSC line (N44SV.5) obtained from Banco Nacional de Líneas Celulares del Instituto de Salud Carlos III (ISCIII, Madrid, Spain). Ethical approval for the use of human samples in the study was granted by the Ethics Committee of the Universidad Autónoma de Madrid and by the authorization of “Dirección General de Investigación, Formación e Infraestructuras Sanitarias”, Community of Madrid, Spain.

iPSCs were cultured in mTeSR^TM^ Plus medium (STEMCELL^TM^ Technologies, Vancouver, BC, Canadá) on plates coated with Matrigel^®^ (Corning, New York, NY, USA) at 37 °C in a humidified atmosphere containing 5% CO_2_. Cells were passaged with ReLeSR™ or with ACCUTASE^TM^ (both from STEMCELL^TM^ Technologies) into a single cell suspension and resuspended in mTeSR^TM^ Plus with 10 µM ROCK Inhibitor (STEMCELL^TM^ Technologies).

### 3.2. Cardiomyocyte Differentiation

Cardiac differentiation was induced in RPMI/B27 medium with Wnt/β-catenin inhibitors, as described previously [42]. Briefly, iPSC colonies were harvested after ACCUTASE^TM^ treatment and seeded onto Matrigel^®^-coated 12-well plates at a density of 5 × 10^5^ cells/well of control iPSCs and 1 × 10^6^ cells/well of *PCCA* iPSCs. On day 0, the cells were treated with 12 µM CHIR-99021 (Selleck Chemicals) in insulin-free RPMI/B27 medium for 24 h. The medium was replaced with basal medium for another two days. On day 3, the culture medium was replaced with 5 µM IWP-4 (Stemgent) insulin-free RPMI/B27 for 48 h. On day 7, the culture medium was changed to RPMI/B27 containing insulin, and the culture medium was refreshed thereafter every two days.

### 3.3. Immunostaining

Cardiomyocytes were seeded onto Matrigel^®^-coated 15 µ-Slide 8 well culture plates (Ibidi, Gräfelfing, Germany), fixed with Formalin Solution 10% (Sigma-Aldrich, St. Louis, MO, USA), and stained with Anti-Troponin T (1:200; cTNT, Sigma-Aldrich), anti-GATA4 (1:50; GATA4, Santa Cruz Biotechnology, Dallas, TX, USA), α-Smooth Muscle Actin (1:250; SMA, Sigma-Aldrich) and Anti-α-Actinin (1:200; α-ACT, Sigma-Aldrich). Alexa Fluor dye secondary antibodies were used (1:200). Microscopic images were obtained using a Zeiss Confocal Fluorescence Microscope.

### 3.4. Flow Cytometry

Cardiomyocytes were trypsinized, washed with PBS and dead cell stained following the LIVE/DEAD™ Fixable Near-IR Dead Cell Stain supplier’s instructions. Cells were washed and fixed with 10% formalin for 20 min. Subsequently, cells were permeabilized with PBS-0.2% Tween for 15 min, washed and incubated overnight at 4 °C with Anti-Troponin T (1:200). The next day, cells were washed and incubated with the secondary antibody Alexa Fluor^®^ 647 (1:750) for 30 min at 4 °C. Finally, cardiomyocytes were washed and analyzed using a FACSCanto A (Becton Dickinson, Franklin Lakes, NJ, USA) and the FlowJo 10.7.0 software program. Unstained cells and the corresponding isotype antibodies were used as negative controls to exclude data from nonspecific fluorescence.

### 3.5. mRNA and miRNA Analysis

Total RNA was extracted using miRNeasy Mini Kit (QIAGEN, Hilden, Germany) according to the manufacturer’s instructions. Concentration and integrity of total RNA was measured in the NanoDrop ND-1000 spectrophotometer (NanoDrop Technologies Inc., Rockland, DE, USA). cDNA was obtained by retrotranscription of 500 ng of total RNA using NZY First-Strand cDNA Synthesis Kit (NZYTech, Lda, Lisbon, Portugal) for mRNA analysis; and of 5 ng of total RNA using the miRCURY LNA RT Kit (QIAGEN) for miRNA analysis. Genes and miRNAs were amplified with specific primers (available upon request) using PerfeCTa SYBR Green FastMix kit (Quanta Biosciences, Beverly, MA, USA) for mRNA analysis; and using the miRCURY LNA SYBR Green PCR Kit (QIAGEN) for miRNA analysis in a LightCycler 480 II instrument (Roche Life Science, Penzberg, Germany), according to the manufacturer’s instructions. For RNA analysis, GAPDH was used as an endogenous control, and miR-423-3p and snRNA U6 were used for normalization in miRNA analysis. Relative mRNA and miRNA expression was quantified using the comparative threshold method after the detection of the different *Ct* values using the 2^−ΔΔ^*^Ct^* method. All samples were run in triplicate.

### 3.6. Immunoblotting

Whole-cell protein extract of cardiomyocytes were made from frozen pellets by lysis performed by freeze-thawing in a buffer containing Tris HCl pH 7.4, 10% glycerol, 150 mM NaCl, 0.1% Triton X-100 and Protease and Phosphatase Inhibitor Cocktail (Sigma-Aldrich), and centrifuged 10 min at 4 °C. The supernatant fraction was collected, and protein concentration was determined by the Bradford method (Bio-Rad Laboratories, Hercules, CA, USA).

For western blot analysis, equal amounts of protein (50–75 µg) were loaded into 4–12% NuPAGE™ Precast Gels or 10% NuPAGE™ Precast Gels. After electrophoresis, proteins were transferred to a nitrocellulose membrane in an iBlot Gel transfer device (Invitrogen, Carlsbad, CA, USA). Immunodetection was carried out using commercially available antibodies against *PCCA* (1:250, Santa Cruz Biotechnology), HERP (1:100, Enzo Life Science, Farmingdale, NY, USA), GRP78 (1:1000, Novus Biologicals, Centennial, CO, USA), GRP75 (1:1000, Abcam, Cambridge, UK), SIG-1R (1:1000, Santa Cruz Biotechnology), MFN2 (1:1000, Abcam), S6 (1:1000, Cell Signaling Technology, Danvers, MA, USA) and phosphorylated S6 (1:1000, Cell Signaling). Anti-mouse IgG HRP-linked (1:2000, Cell Signaling) and anti-rabbit IgG HRP-linked (1:5000, Cell Signaling) were used as secondary antibodies. Antibody against GAPDH was used as a loading control (1:5000, Abcam). Enhanced chemiluminescence reagent (ECL, GE Healthcare, Chicago, IL, USA) was used for protein detection. Band intensity for each protein was quantified with a BioRad GS-900 Densitometer (Bio-Rad, Hercules, CA, USA) and the ImageLab program.

### 3.7. Electron Microscopy

Cardiomyocytes seeded in p35 plates with Matrigel^®^ and RPMI/B27 containing insulin, 10% FBS and 10 µM Y27632 were fixed with 4% paraformaldehyde and 2% glutaraldehyde in 0.1 M phosphate buffer pH 7.4 for 2 h at room temperature. Fixed cells were included in epoxy resin (TAAB 812 resin, TAAB laboratories, Berkshire, England) by conventional methods. For this, cells were stained for 1 h with 1% osmium tetroxid + 0.08% potassium ferricianide at 4 °C and with 2% uranyl acetate in water for another hour at 4 °C. Dehydration (EtOH: 50%, 75%, 90%, 95% and 100%) was carried out at 4 °C and embedded with the resin (epoxy resin: EtOH 1:2, 1:1, 2:1, and 100% resin) at room temperature. Finally, the resin was polymerized for 48 h at 60 °C. Ultrathin 70 nm cuts were obtained on a Leica Ultracut UCT ultramicrotome (Leica, Vienna, Austria) with a DiATOME diamond blade. Sections were collected on hexagonal drawing Cu/Pd grids, and 100 windows per square inch (200 mesh) were coated with formvar and a layer of evaporated carbon. The sections were stained with 2% uranyl acetate in water for 7 min and with Reynolds lead citrate for 3 min. About 100 images from each sample were taken with a 4 K × 4 K, F416 CMOS camera from TVIPS (Gauting, Germany) at 5000× or 8000× magnification on the JEOL JEM-1010 electron microscope (JEOL, Akishima, Tokyo, Japan) at an electron acceleration voltage of 80 kV.

### 3.8. Extracellular Flux Assay

The cellular oxygen consumption rate (OCR) was measured using an XF24 Extracellular Flux Analyzer (Seahorse Bioscience, Agilent Technologies, Santa Clara, CA, USA). At 72 h before the assay, 0.06 × 10^6^ cells per well were seeded in XF 24-well cell culture microplates coated with Matrigel^®^ in a total volume of 100 µL in RPMI/B27, 10% fetal bovine serum (FBS) and 10 µM ROCK Inhibitor. One hour later, an additional 150 µL of medium was added to each well. After 48 h, the medium was changed to 250 µL of MEM medium with 10% FBS and the calibration plate was hydrated with Seahorse XF Calibrant solution overnight at 37 °C. One hour before the assay, the growth medium was replaced with 700 µL of unbuffered fresh MEM medium with 0.5% FBS. After taking an OCR baseline measurement, 50 µL of oligomycin, carbonyl cyanide-4-(trifluoromethoxy) phenylhydrazone (FCCP), rotenone and antimycin A solutions were sequentially added to each well to reach final working concentrations of 2, 1.5, 2 and 2 µM, respectively. Basal respiration was measured without substrates. Oxygen consumption coupled to ATP production (ATP-linked) was calculated as the difference between basal respiration and the proton leak state determined after the addition of oligomycin. Maximum respiration was measured by stepwise 1.5 µM titration of FCCP and inhibition by rotenone and antimycin. Spare capacity was calculated as the difference between the maximum and basal respiration. The results were normalized to the protein amount and analyzed by using Seahorse XF24 software.

### 3.9. Statistical Analysis

All values shown were average values from *n* experiments that were carried out independently and with different biological samples. Cardiomyocyte differentiation from iPSC lines was performed 10 times, and the analyses were carried out with at least three biological replicas for triplicates. The statistical significance of the differences between the analyzed groups was evaluated using a two-tailed unpaired *t*-test distribution. The differences were considered significant based on the obtained *p*-values: * <0.05, ** <0.01 and *** <0.001.

## Data Availability

The data that supported the findings of the present study are available from the corresponding author upon request.

## Abbreviations

α-ACT	α-Actinin
CRISPR	Clustered regularly interspaced short palindromic repeats
cTnT	Cardiac troponin T
ER	Endoplasmic reticulum
*FBL*	rRNA methyltransferase fibrillarin
GRP75	75-kDa Glucose regulated protein
GRP78	78-kDa Glucose-regulated protein
HERP	Homocysteine-inducible ER stress protein
iPSCs	Induced pluripotent stem cells
iPSC-CMs	iPSC-derived cardiomyocytes
MAMs	Mitochondria-associated membranes
MFN2	Mitofusin-2
*NCL*	Nucleolin
OA	Organic acidemia/aciduria
OCR	Oxygen consumption rate
PA	Propionic acidemia
PBS	Phosphate buffered saline
PCC	Propionyl-CoA carboxylase
*POLR1A*	Large subunit of Pol I
PPAR	Peroxisome proliferator activated receptors
qRT-PCR	Real-time quantitative reverse transcription- polymerase chain reaction
*RRN3*	pol I-pecific transcription initiation factor
SIG-1R	Sigma-1 receptor
*SIRT7*	Pol I-activating NAD-dependent histone deacetylase sirtuin 7
SMA	Alpha-smooth muscle actin
*UBTF*	Ribosomal RNA upstream binding transcription factor
UPR	Unfolded protein response
WT	Wild-type
